# Looking for a strategy in treating peritoneal gastric cancer carcinomatosis: an Italian multicenter Gastric Cancer Research group’s analysis

**DOI:** 10.1186/s12957-021-02442-9

**Published:** 2021-11-24

**Authors:** Luigina Graziosi, Elisabetta Marino, Maria Bencivenga, Alessia D’Ignazio, Leonardo Solaini, Silvia Ministrini, Michela Caprioli, Michele Sacco, Daniele Marrelli, Gianni Mura, Maurizio Degiuli, Paolo Morgagni, Guido Alberto Massimo Tiberio, Giovanni De Manzoni, Franco Roviello, Annibale Donini

**Affiliations:** 1 General and Emergency Surgery, Santa Maria della Misericordia Hospital University of Perugia, Perugia, Italy; 2 General and Upper GI Surgery Division, University of Verona, Verona, Italy; 3 Department of Surgery, Policlinico le Scotte, University of Siena, Siena, Italy; 4 Department of Surgery, General and Oncologic Surgery, Morgagni-Pierantoni Hospital, Via Forlanini 34, Forlì, Italy; 5 Department of Experimental and Clinical Sciences, University of Brescia, Brescia, Italy; 6 Department of Surgery, Azienda USl Toscana SudEst—Arezzo, Arezzo, Italy; 7 Department of Oncology, Head, Digestive and Surgical Oncology, University of Torino, and San Luigi University Hospital, Orbassano, Italy

**Keywords:** Gastric cancer, Peritoneal carcinomatosis, Surgery

## Abstract

**Background:**

The present study provides a snapshot of Italian patients with peritoneal metastasis from gastric cancer treated by surgery in Italian centers belonging to the Italian Research Group on Gastric Cancer. Prognostic factors affecting survival in such cohort of patients were evaluated with the final aim to identify patients who may benefit from radical intent surgery.

**Methods:**

It is a multicentric retrospective study based on a prospectively collected database including demographics, clinical, surgical, pathological, and follow-up data of patients with gastric cancer and synchronous macroscopic peritoneal metastases. Patients were surgically treated from January 2005 to January 2017. We focused on patients with macroscopic peritoneal carcinomatosis (PC) treated with upfront surgery in order to provide homogeneous evidences.

**Results:**

Our results show that patients with peritoneal carcinomatosis cannot be considered all lost. Strictly selected cases (R0/R1 and P1 patients) could benefit from an aggressive surgical approach performing an extended lymphadenectomy and HIPEC treatment.

**Conclusion:**

The main result of the study is that GC patients with limited peritoneal involvement can have a survival benefit from a surgery with “radical oncological intent”, that means extended lymphadenectomy and R0 resection. The retrospective nature of this study is an important bias, and for this reason, we have started a prospective multicentric study including Italian stage IV patients that hopefully will give us more answers.

## Background

In spite of early diagnosis and improved treatments, gastric cancer (GC) remains the fifth leading cause of tumor-related death worldwide [1]. The lack of screening programs in the West that leads to late diagnosis and high rate of postoperative recurrences is one of the main reasons of such poor prognosis.

Although new chemotherapy strategies have been recently introduced in clinical practice, metastatic and recurrent GCs show a dramatic median survival time (MST) of only 3–9 months [2–7].

Stage IV GC consists of heterogeneous conditions including hematogenous metastases, distant lymph node metastasis, peritoneal carcinomatosis, or even a mixture of them.

Recently, Yoshida et al. [8] suggested new categories for stage IV GC based on oncosurgical treatment strategies. In addition, he clarified the definitions of *conversion therapy* as a surgical treatment aiming at a complete surgical resection (R0) after chemotherapy of metastatic gastric tumors that were originally considered as technically and/or oncologically unresectable.

A common site of metastasis in gastric cancer is the peritoneal cavity; indeed approximately 15% of patients diagnosed with primary GC, show synchronous peritoneal carcinomatosis (PC) [9–12].

Moreover, Yang et al. observed that a high percentage, up to 52.4%, of patients with advanced gastric cancer, even after a macroscopically curative D2 gastrectomy followed by adjuvant chemotherapy, showed PC as a single pattern of recurrence [13].

Both synchronous and metachronous PC are associated with a very bad survival of approximately 2–4 months.

Serosa involvement, diffuse histotype, and proximal location are risk factors for PC [14].

In Western countries, different epidemiological trends were observed during the last decades. Tumors located in the distal third of the stomach are decreasing in favor of locally advanced proximal and diffuse-type tumors [15–17] with a higher risk of peritoneal dissemination.

As such, there is an increasing interest to further improve survival outcomes in stage IV GC patients, especially of those affected by PC.

Recently, the Italian Research Group for Gastric Cancer guidelines (GIRCG) [18] stated that some patients with unresectable stage IV GC could benefit from intensive combined treatments including radical surgery after first-line chemotherapy achieving long-term survival.

The literature also reinforced the concept that conversion surgery for unresectable stage IV gastric cancer, including peritoneal involvement, was associated with longer survival than chemotherapy alone ranging from 37 to 56 months [19–23].

According to recent evidences, cytoreductive surgery (CRS) plus hyperthermic intraperitoneal chemotherapy (HIPEC) could represent a promising multidisciplinary approach for a selected subgroup of GC patients with limited peritoneal carcinomatosis (PC) when an apparently R0 resection can be achieved [24, 25].

Currently, new trials are ongoing to prove the effectiveness of such strategies (GASTRICHIP trial and German phase II HIPEC-Stomach trial).

The present study provides a snapshot of Italian patients with PC from gastric cancer treated by surgery in centers that used to work with the same guidelines [18]. Then we evaluated prognostic factors affecting survival in such cohort of patients with the final aim to identify PC patients who may benefit from radical intent surgery.

## Methods

### Population and study design

The present is a multicentric retrospective study based on a prospectively collected database including demographics, clinical, surgical, pathological, and follow-up data of 166 patients with gastric cancer and synchronous macroscopic peritoneal metastases or positive peritoneal cytology; all patients were surgically treated from January 2005 to January 2017 at seven institutions belonging to the Italian Research Group on Gastric Cancer and managed with patient consent according to the singular institutions. Eight patients were excluded due to missing data or due to an emergency (occlusion or bleeding setting) surgery.

**Fig. 1:**
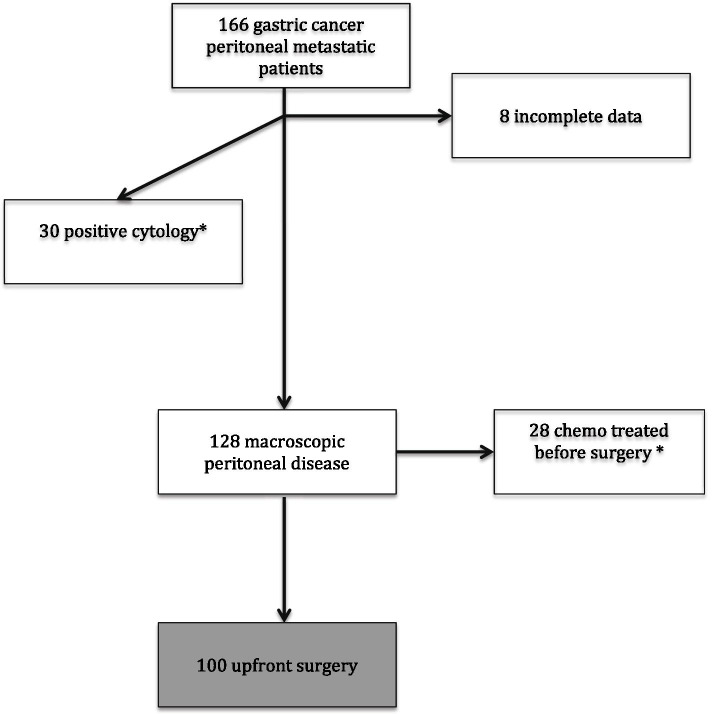
Flowchart of the patients enrolled in the study; *data will be presented in another paper

Our study is focused on patients with macroscopic PC treated with upfront surgery in order to provide homogeneous evidences, as such, 30 patients with only positive cytology and 28 patients that underwent chemotherapy before surgery were excluded; data of these former categories will be analyzed separately and presented in the future. Finally, 100 patients were enrolled in the study as shown in Fig. 1.

The mean age of our population was 68.49 ± 12.17; male to female ratio was 64/36.

Ten percent of patients in our series showed an upper-third tumor location, 38% a middle-third location, 47% a lower-third location, and 5% had plastic linitis.

All the patients included in the study, in accordance to the singular institutions policy, signed a written consent.

All procedures followed were in accordance with the ethical standards of the responsible committee on human experimentation (institutional and national) and with the Helsinki Declaration of 1964 and later versions.

### Diagnosis and follow-up

The diagnosis of peritoneal carcinomatosis in the preoperative setting was performed by contrast enhancement tomography (CETC) scan and peritoneal disease grade was defined according to Japanese classification of gastric carcinoma 2nd edition [26].

Results were reported by the expert reader to identify serosa invasion and direct or indirect markers of peritoneal involvement. Staging laparoscopy was integrated in diagnostic and staging programs but was not performed in all patients because it was not yet included as a standard procedure in every institution. In detail, 6 staging laparoscopies (6.0%) were done in the enrolled patients. Therefore, peritoneal involvement was sometimes diagnosed during the main surgery.

After surgery, follow-up was carried out every 3 months for the first 2 years and once a year afterward for another 8 years. Follow-up was based on clinical evaluation, CECT, measurement of tumor markers (CEA and CA19-9), and upper endoscopy. All the evaluations were discussed within a multidisciplinary team made up of surgeons, clinical oncologists, radiotherapists, and radiologists.

### Surgery and staging classification

All patients underwent distal or total gastrectomy. Total omentectomy was usually integrated in the standard gastrectomy. When the posterior gastric wall serosa was infiltrated by the tumor, peritoneal surface of the bursa omentalis was resected, too. Standard lymphadenectomy was considered D2, but in selected cases such as patients with serious comorbidities, and/or with an advanced age, more limited lymphadenectomy (D1 or D1+) was performed. In case of a total gastrectomy, station 10 lymphadenectomy was performed only when the tumor was involving the greater curvature and/or the posterior wall of the stomach. In these cases, the spleen was preserved. In patients with high risk of distal lymphododal spreading (advanced tumors of the upper third, advanced tumors and diffuse histotype located in the distal two thirds of the stomach, bulky nodes), D2+ or a lymphadenectomy extended to posterior and PAN stations was performed.

Cytoreductive surgery (CRS) plus HIPEC was reserved to patients with limited peritoneal involvement (P1, P2) in which a R0 could be likely achieved. The technique was standardized between each GIRCG centers. Most of the patients underwent limited peritonectomies, and few cases underwent mutivisceral resection, in particular distal splenopancreasectomy and Krukemberg disease removal.

HIPEC was carried out with Cisplatin and mytomicin C.

Tumor stage was presented as indicated by the Union for International Cancer Control (UICC)/American Joint Committee on Cancer (AJCC) 8th edition. Curative surgery was defined when R0 resection was performed according to the residual tumor classification [27].

Other clinical and pathological characteristics of the patients are summarized in Table 1.

**Table 1:** Patients’ clinicopathological characteristics

Characteristics	***N*** = 100
**Age**^**a**^	68,49 (62–78)
**Gender**	
**M**	64 (64%)
**F**	36 (36%)
**Tumor location**	
**Upper**	10 (10%)
**Body**	38 (38%)
**Lower**	47 (47%)
**Linitis**	5 (5%)
**Gastrectomy**	
**Total**	42 (42%)
**Subtotal**	58 (58%)
**Lymphadenectomy**	
**D1**	36 (36%)
**D2**	46 (46%)
**+**	18 (18%)
**HIPEC**	
**Yes**	11 (11%)
**No**	89 (89%)
**Adjuvant chemotherapy**^**b**^	
**Yes**	42 (42%)
**No**	35 (35%)
**Surgical radicality**^**b**^	
**0**	38 (38%)
**1**	18 (18%)
**2**	43 (43%)
**pT**	
**1**	0
**2**	2 (2%)
**3**	32 (32%)
**4a**	54 (54%)
**4b**	12 (12%)
**pN**	
**0**	2 (2%)
**1**	7 (7%)
**2**	14 (14%)
**3**	71 (71%)
**x**	6 (6%)
**Lymph nodes harvested**^**b**^	40.85 (28–53)
**Peritoneal involvement**^**b**^	
**P1**	34 (34%)
**P2**	17 (17%)
**P3**	23 (23%)
**Lauren’s type**^**b**^	
**Intestinal**	37 (37%)
**Diffuse**	46 (46%)
**Mixed**	16 (16%)

^a^Indicated as mean and 25th–75th percentile of value

^b^Some data are missing

### Statistical analysis

Descriptive statistics are presented as median or average and interquartile range (IQR 25–75%) or standard deviation. Comparisons between groups were obtained with the chi-squared analysis for discrete variables, whereas Student’s *t* test analysis was utilized for continuous variables. Overall survival (OS) was measured from the date of resection to the date of death or the latest follow-up. Survival analyses were generated according to the Kaplan–Mayer method, and statistical significance was determined using the log-rank test. All the variables were than considered for multivariate analysis with Cox proportional hazards model; a *p* < 0.05 was considered statistically significant.

## Results

**Fig. 2:**
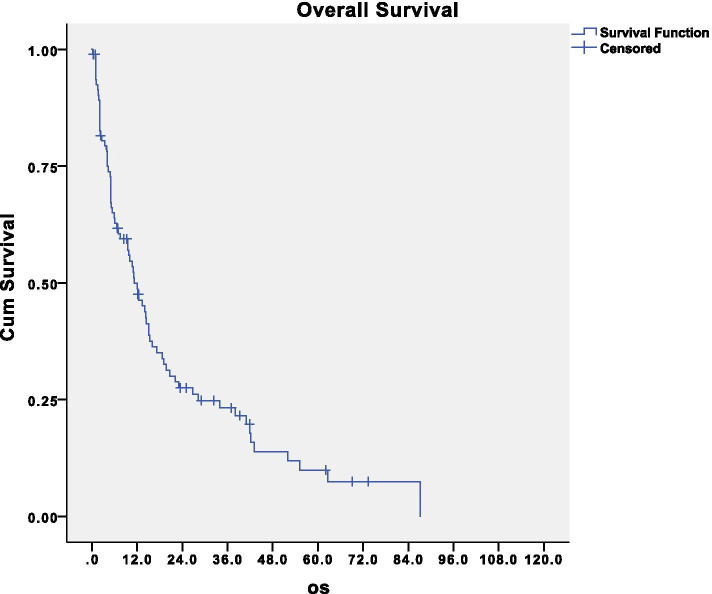
Disease-related survival of the entire population analyzed

The median survival was 11.2 months, and the 5-year overall survival was 10% (Fig. 2). After an immediate sharp decline in survival, a 3-year survival of about 24% was shown, and also 5-year survivors were observed (10%).

Tumor site and type of surgery (total *vs.* subtotal gastrectomy, 42% vs. 58%) had no significant impact on disease-related survival.

**Fig. 3:**
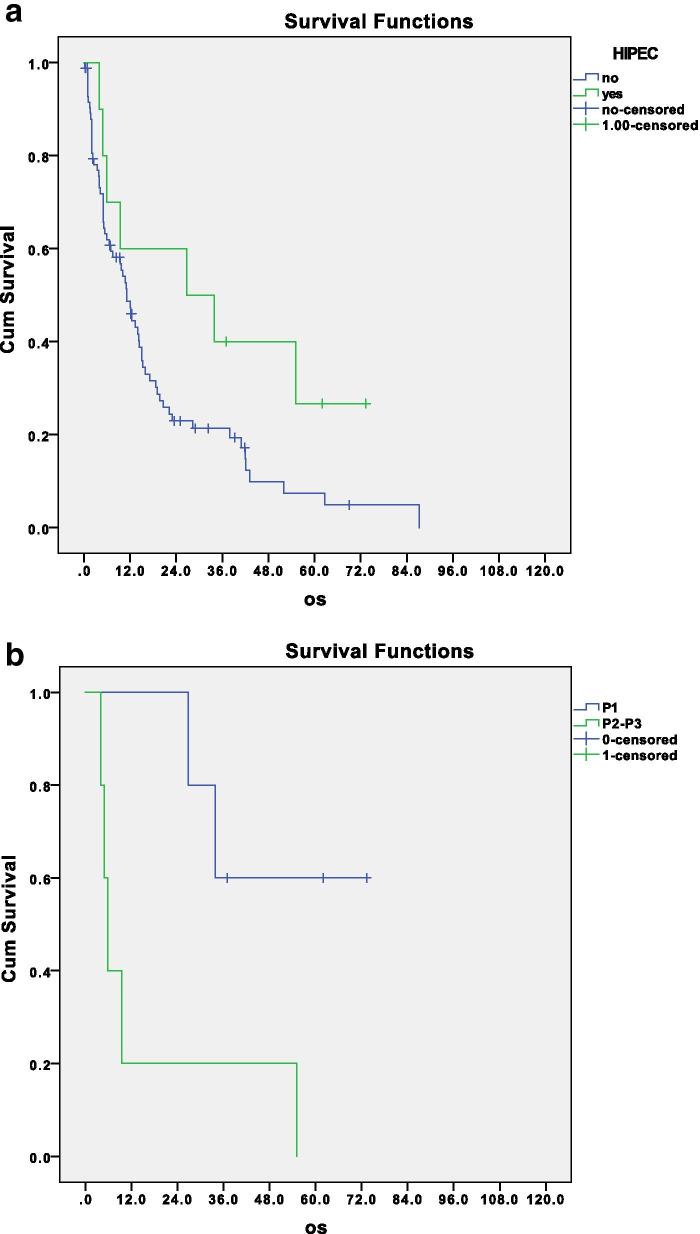
**a** Disease-related survival of patients with macroscopic disease according to HIPEC treatment, *p* = 0.07; **b** disease related survival of patients treated with HIPEC according to the peritoneal involvement, *p* = 0.02

Of note, the use of HIPEC showed a trend in improving survival of these patients, even if a statistical significance was not reached. The median survival of patients that underwent HIPEC treatment was 26.8 months versus 11.2 months of patients who did not receive HIPEC (*p* = 0.07) (Fig. 3a).

This may reflect a bias by indication, as HIPEC was more frequently performed in patients with p1 PC (*n* = 5). Comparing HIPEC treatment in patients with a localized disease (p1), versus patients with a more advanced peritoneal involvement (p2–p3; *n* = 5), the survival improvement was significant as shown in Fig. 3b (*p* = 0.02). This stresses the concept of the selected use of HIPEC in patients with limited peritoneal involvement and total surgical resection.

**Fig. 4:**
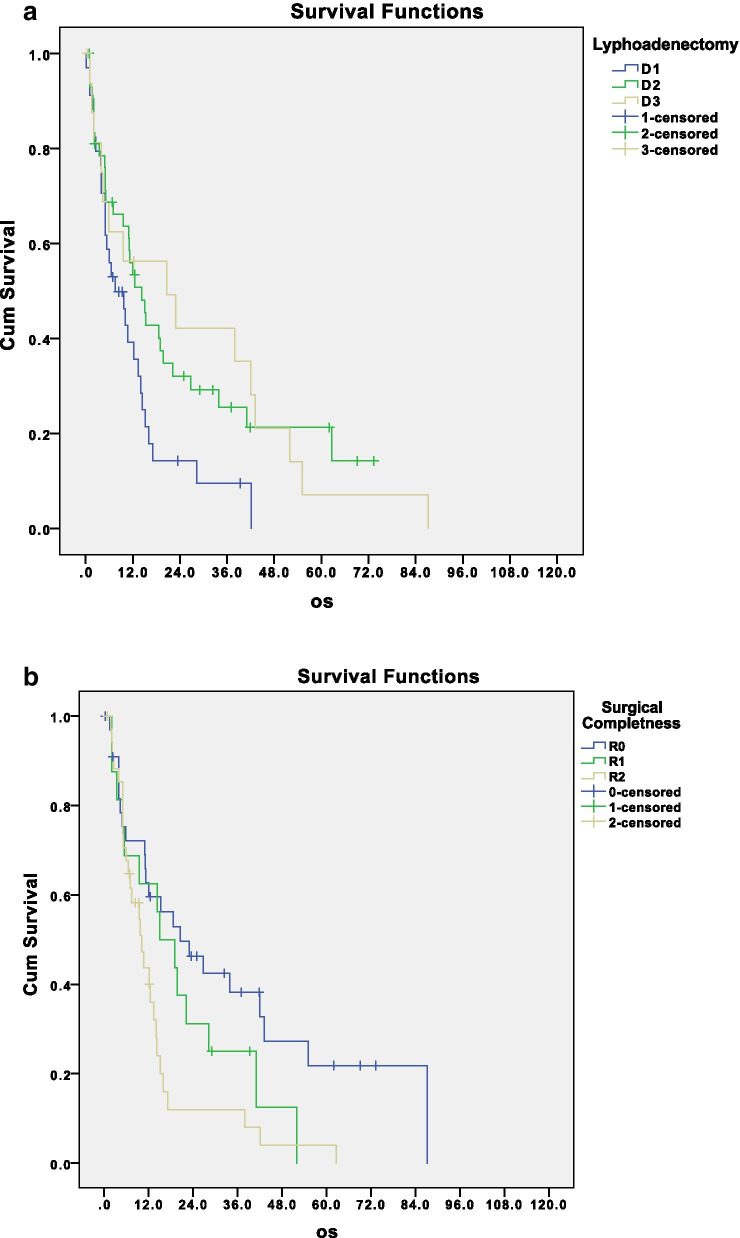
**a** Disease-related survival according to lymphadenectomy extension, *p* = 0.04; **b** disease-related survival according to completeness of surgical resection, *p* = 0.01

D1 lymphadenectomy was performed in 36% of patients, D2 in 48%, and D2+ in 18% of patients. Of note, the type of lymphadenectomy (D1, D2, or plus) significantly influenced disease-related survival of patients treated with upfront surgery. Median survivals of patients that underwent D1 or D2 or D3 lymphadenectomy were respectively 10.1, 15.3, and 23.0 months as shown in Fig. 4a (*p* = 0.03). This is another debated issue, emphasizing the crucial role of lymphadenectomy also in advanced disease; the surgeon should not avoid a superextended lymphadenectomy in front of the peritoneal disease, when he/she is able to perform a complete cytoreduction.

R0/R1 resection was achieved in 56% of patients; 43% of patients had macroscopic residual disease.

According to pT, patients were stratified as follow: 2% pT2, 32% pT3, 54% pT4a, and 14% pT4b. According to pN, 2% were N0, 7% N1, 14% N2, 71% N3, and 6% Nx due to the lower number of nodes retrieved.

**Fig. 5:**
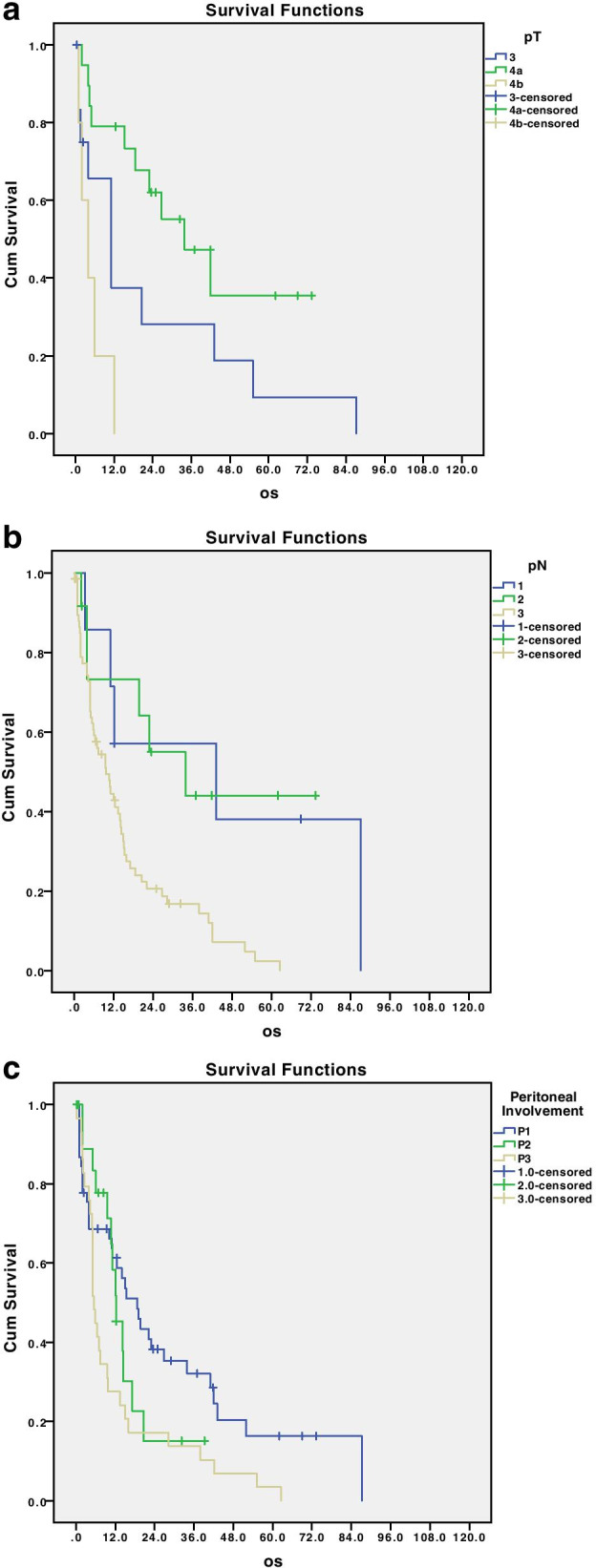
Disease-related survival according to pathological T stage (**a**), pathological N stage (**b**), and peritoneal involvement (**c**); *p* = 0.002, 0.003, and 0.023, respectively

As expected, completeness of surgical resection (Fig. 4b), pT, pN, and the grade of peritoneal involvement were shown as significant risk factors for overall survival (Fig. 5a, b, and c; *p* = 0.002; *p* = 0.003; *p* = 0.023).

The median number of harvested lymph nodes was 31; however, neither dichotomizing patients for this value, nor for the average value of lymph nodes harvested (*n* = 35) in the entire series had a significant prognostic role for overall survival.

Nevertheless, the median survival in the patients with a higher number of harvested lymph nodes seems to have a better trend compared with the ones with a lower number of harvested nodes especially in R0/R1 subgroups.

Lauren subtypes did not seem to impact the disease-free survival.

Adjuvant chemotherapy performed only in 42% of patients did not add any statistically significant survival benefits in this subgroup of patients even though there is a trend in favor of those patients that underwent chemotherapy.

At the multivariate analysis, the independent prognostic factors for overall survival were extension of peritoneal involvement, type of lymphadenectomy, and surgical radicality as shown in Table 2 with a *p* < 0.05.

**Table 2:** Univariate and multivariate analysis of patients with macroscopic peritoneal involvement treated with upfront surgery

Variable	Univariate	Multivariate	
		HR	***P***
**Age**			
**> 68 (ref.)**	0.33	1.04	0.59
**< 68**			
**Gender**			
**Female**	0.22	1.17	0.80
**Male (ref)**			
**Tumor location**			
**Upper (ref.)**			
**Body**	0.53	1.03	0.10
**Antrum**			
**Surgery type**			
**Subtotal gastrectomy**			
**Total gastrectomy (ref.)**	0.36	2.35	0.07
**Lymphadenectomy**			
**D1 (ref.)**			
**D2**	0.04	2.76	**0.03**
**PLUS**			
**pT**			
**3**			
**4a (ref.)**	0.002	2.14	0.12
**4b**			
**pN**			
**N1 (ref)**			
**N2**	0.003	1.49	0.3
**N3**			
**Lauren’s istotype**			
**Intestinal**	0.56	2.35	0.08
**Diffuse**			
**HIPEC**			
**Yes**	0.07	0.34	0.11
**No**			
**Surgical radicality**			
**R0 (ref.)**			
**R1**	0.01	1.68	**0.03**
**R2**			
**Peritoneal involvement**			
**P1 (ref)**			
**P2**	0.023	1.03	**0.03**
**P3**			
**Adjuvant chemotherapy**			
**Yes**	0.34	1.17	0.73
**No**			

## Discussion

GC peritoneal carcinomatosis is a fatal disease impacting dramatically patients’ survival. The median overall survival (OS) has remained to be less than 1 year despite the introduction of new chemotherapies [28].

Recently, numerous and various modalities of treatment have been tried to approach GC peritoneal metastasis (PM), including aggressive surgery, intraperitoneal hyperthermic chemotherapy (HIPEC), extensive peritoneal lavage (EIPL) and chemotherapy alone, but none has provided to date satisfactory clinical outcomes [29–32].

Consequently, to date there is not a standardized treatment for patients with PC.

In the Regatta Trial, peritoneal metastasis was the most common non-curable factor in 75% of all oligometastatic patients; the authors asserted that palliative surgery did not improve the OS, leaving chemotherapy alone as the standard of care for these patients. Regatta does not completely exclude the possibility of gastrectomy in oligometastatic stages of GC but highlights the necessity of an optimal timing in the setting of a combined treatment approach [33].

On the other hand, Thomassen et al. [34] highlighted that chemotherapy did not prolong survival of patients with PC from gastric origin. Therefore, the beneficial effect of current chemotherapy regimens remains questionable at least in this patient category, and its effectiveness has been virtually absent during the years.

It is hypothesized that the effect of intravenous chemotherapy on peritoneal metastases is limited due to the peritoneal blood barrier [35].

Recent evidences suggest that EIPL with a large volume (at least 10 L) of normal saline after surgery before abdominal closure can reduce the risk of peritoneal recurrence and improve overall survival in patients at high risk of PC. To date, we are waiting for the results of a randomized controlled trial (RCT), which assessed the potential effects of EIPL in preventing PM after curative surgery in patients with serosa involvement or positive cytology [36].

Neoadjuvant intraperitoneal and systemic chemotherapy (NIPS) is the current conversion bidirectional therapy for GC patients with peritoneal metastasis. The meta-analysis recently made by Yingbo et al. [37] showed the effectiveness and safety of NIPS combined to surgery for GC patients with PM but much higher quality trials and multicenter randomized controlled trials are needed to firstly demonstrate the real benefit and then to support this aggressive treatment in oncological guidelines.

However a multimodal approach including CRS and HIPEC remains the main strategy in Western Countries as stated in the GIRCG and French guidelines [18, 26], respecting limited inclusion criteria in terms of the peritoneal extension.

In the past, Eastern data have reinforced this multimodality approach: a well done systematic review and meta-analysis of 13 acceptable-quality randomized controlled trials has established that HIPEC is associated with a marked improvement in survival in advanced GC, in comparison with the current standard treatments [38].

In 2003, an international panel of major experts in peritoneal disease strongly recommended that CRS plus HIPEC could be the current standard treatment for GC with PC [39].

Coccolini et al. reinforced in his meta-analysis the important role in improving OS of GC patients showing PC [40].

Nevertheless, controversy over this treatment modality remains.

PHOENIX-GC trial results were recently published, and they failed to show superiority of intraperitoneal chemotherapy than systemic chemotherapy. However, the authors concluded their work assessing that after an exploratory analysis, possible clinical benefits were given by intraperitoneal paclitaxel. It could be explained by the fact that the combination of neoadjuvant chemotherapy according to the FLOT scheme with a cytoreductive surgery plus HIPEC and subsequent re-systemic therapy can increase the median survival to at least 17 months [41].

Our study results as the GYMSSA trial [42] emphasized the positive impact of the multimodality therapy combining CRS plus HIPEC compared with only systemic chemotherapy on OS in selected patients affected by gastric carcinomatosis with limited burden of disease.

The main findings of the present study are that patients with gastric cancer and limited synchronous PC do not have negligible long-term survival when treated with aggressive surgery including extended lymphadenectomy and HIPEC. These results would surely improve in the context of a multimodal pre or perioperative intensive chemotherapy. According to the previous GIRCG study [43], we underlined the importance of an R0 cytoreductive surgery that could give a survival benefit and a possibility of an effective cure also in stage IV patients.

We can conclude asserting that metastatic gastric cancer is still a challenge for everybody and in particular for the oncologist surgeon. Specifically, peritoneal metastasis is a field in which surgeons, can play an important role in a selected group of patients.

In the near future, molecular analysis will hopefully allow a proper selection of patients in this clinical setting.

Moreover, the Yoshida categories [8], which do work for the Eastern countries and pathology, may not fit our Western cases, forcing us to continue to study insight of the neoplasm and stressing once again the intrinsic differences in gastric cancer pathology.

## Conclusion

Our results show that patients with peritoneal carcinomatosis cannot be considered all lost. Certainly in some selected cases (R0/R1 and P1 patients), they could benefit from an aggressive surgical approach performing an extended lymphadenectomy and HIPEC treatment.

The principal limitation is that this is a multicenter and retrospective study involving different centers, but all surgeons are expert in gastric cancer management having a good experience in D gastrectomy and ensuring a high quality of surgery. However, patients were well-staged, and survival rates were similar in each center.

Further studies and prospective ones are needed to better understand GC with PC patients and to bring advancements in therapeutic options considering also molecular patterns according to the recently published molecular classifications of gastric cancer [44], which could result in meaningful improvement in patient survival.

An important bias of our study is its retrospective nature; for this reason, we therefore have started a prospective multicentric study including Italian stage IV patients that hopefully will give us more answers.

## Data Availability

Data will be available upon motived request.
